# The perceived risk of being infected at work: An application of the job demands–resources model to workplace safety during the COVID-19 outbreak

**DOI:** 10.1371/journal.pone.0257197

**Published:** 2021-09-09

**Authors:** Alessandra Falco, Damiano Girardi, Laura Dal Corso, Murat Yıldırım, Daniela Converso

**Affiliations:** 1 Department of Philosophy, Sociology, Education and Applied Psychology, Section of Applied Psychology, University of Padua, Padua, Italy; 2 Department of Psychology, Ağrı İbrahim Çeçen University, Ağrı, Turkey; 3 Department of Neuroscience, Psychology and Behaviour, University of Leicester, Leicester, United Kingdom; 4 Department of Psychology, University of Turin, Turin, Italy; University of East Anglia, UNITED KINGDOM

## Abstract

Safety at work, both physical and psychological, plays a central role for workers and organizations during the ongoing outbreak of COVID-19. Building on the job demands-resources (JD-R) model applied to safety at work, in this study we proposed that the perceived risk of being infected with COVID-19 at work can be conceptualized as a job demand (i.e., a risk factor for work-related stress), whereas those characteristics of the job (physical and psychosocial) that help workers to reduce or manage this risk can be conceived as job resources (i.e., protective factors). We hypothesized that the perceived risk of being infected at work is positively associated with emotional exhaustion. Furthermore, we hypothesized that job resources, in terms of safety systems, communication, decision-making, situational awareness, fatigue management, and participation in decision-making, are negatively associated with emotional exhaustion. We also hypothesized that job resources buffer the association between perceived risk and emotional exhaustion. Overall, 358 workers (mean_age_ = 36.3±12.2 years) completed a self-report questionnaire, and the hypothesized relationships were tested using moderated multiple regression. Results largely supported our predictions. The perceived risk of being infected at work was positively associated with emotional exhaustion, whereas all the job resources were negatively associated with it. Furthermore, safety systems, communication, decision-making, and participation in decision-making buffered the relationship between the perceived risk of being infected at work and emotional exhaustion. In a perspective of prevention and health promotion, this study suggested that organizations should reduce the potential risk of being infected at work, whenever possible. At same time, those characteristics of the job that can help workers to reduce or manage the risk of infection should be strengthened.

## Introduction

COVID-19 is a disease caused by a new coronavirus called SARS-CoV-2. On January 9, 2020, the World Health Organization (WHO) declared that Chinese authorities have made a preliminary determination of a new coronavirus [1]. On March 11, 2020, the WHO announced that COVID-19 can be characterized as a pandemic [2]. In Italy, on February 21, 2020, the Italian National Institute of Health (Istituto Superiore di Sanità) confirmed the first indigenous case [3], and a full national lockdown was then implemented on March 11, 2020 [4]. Since then, COVID-19 has afflicted tens of millions of people in a worldwide pandemic [5], with major health (both physical and mental), social, and economic consequences [6–9]. Furthermore, the COVID-19 outbreak is affecting many areas of everyday life, including family, education, and, of course, work [10–12]. To date, considerable attention has been devoted to health and safety of healthcare professionals at work, who have to handle large numbers of patients and the risk of infecting themselves and others, with considerable consequences in terms of stress and psycho-physical symptoms [13, 14].

However, workers in several occupational sectors are now facing the risk of being infected with COVID-19 at work [12]. Indeed, the workplace and the organization of work have some characteristics that may facilitate the spread of SARS-CoV-2 (although with differences between occupations). These include, for example, physical proximity and close social contact with colleagues or supervisors, working in close, public spaces, as well as frequent social interactions with clients, pupils, or patients [15]. Hence, in the context of the current outbreak of COVID-19, safety at work plays a central role in organizations. Public and private companies need to be productive in a rapidly evolving, pandemic-related scenario (e.g., by adapting to the restrictions imposed by the Government to reduce the spreading of the SARS-CoV-2 virus) while, at the same time, preserving employees’ health and well-being [16]. Interestingly, recent literature on workplace safety suggests that psychosocial safety need to be considered in conjunction with physical safety [17]. With respect to the COVID-19 outbreak, this would imply that, in terms of prevention and health promotion, work-related factors that may lead to both physical and psychological outcomes related to COVID-19 risk (e.g., contracting COVID-19 and work-related stress/job burnout, respectively) need to be carefully considered.

In this study, we propose that the perceived risk of being infected with COVID-19 at work can be conceptualized as a job demand [18]. It can be considered as a risk factor for work-related stress, whereas those characteristics of the job (physical and psychosocial) that help workers to reduce or manage the risk of being infected at work can be conceived as job resources (i.e., protective factors) [19]. First, to thoroughly assess the perceived risk of being infected at work, we adapted the COVID-19 Perceived Risk Scale (CPRS) [20], an instrument originally aimed at assessing perceived risk in the general population, to the work context. Then, building on previous research on the job demands–resources (JD-R) model [21–23] applied to safety at work [17, 24, 25] we investigated the association between perceived risk and emotional exhaustion, a core component of job burnout [26]. We also examined the association between job resources related to safety at work and emotional exhaustion, as well as the moderating role of these job resources in the relationship between perceived risk and emotional exhaustion. Finally, given that the JD-R focuses on the relationships between classes of constructs (e.g., job demands and impaired health) without giving a detailed explanation of the underlying theoretical mechanisms [22, 27], in the discussion section of the article we also build on the conservation of resources (COR) theory [28–30] to justify the proposed relationships. COR is a motivational theory whose central assumption is that individuals are motivated to protect their current resources and acquire new resources, that is, things (e.g., objects, conditions, or energies) that people value [31]. Generally speaking, building on the COR we argue that the perceived risk of being infected at work may threaten resources of crucial importance to individuals, such as health and work-related safety, whereas job resources related to safety at work may help individuals to protect these resources. We elaborate on these arguments in the discussion section.

### The perceived risk of being infected at work

Risk refers to both the probability of being harmed as well as the severity and quality of the harmful consequences, should they occur [32]. Although different perspectives exist in the literature [33], risk can be conceptualized as a complex, socially-constructed phenomenon that is defined by individuals, who may be affected in the process by psychological, social, and cultural factors [34–36]. Not surprisingly, extensive research has been carried out on risk perception and perceived risk. Risk perception has been conceived as the "subjective assessment of the probability of a specified type of accident happening and how concerned we are with such an event" (p.152) [37]. Accordingly, risk perception involves not only sensory perception, but also individuals’ attitudes and expectations [32]. At least two dimensions of risk perception have been identified in the literature, namely a cognitive component and an emotional or affective component [38, 39]. Hence, risk perception may be described as a multidimensional construct that encompasses both the perceived likelihood of experiencing an accident, injury, or harmful consequences caused by exposure to a risk source as well as affective reactions to the source, which may include the assessment of worry, concern, or feelings of safety [38–41].

Several previous studies considered both the cognitive and affective/emotional aspects of risk perception, including research carried out in the field of risky driving behaviour [42], risky riding behaviour [43], work-related accident [44], natural disasters such as hurricanes [45], and climate change [46]. Similarly, an analogous approach was also adopted in studies on risk perceptions related to SARS and Avian influenza [47, 48] as well as Swine influenza [49]. Recently, researchers worldwide devoted attention to the perceived risk related to COVID-19 infection [20, 50–57], showing that perceived risk was associated with negative affect and negative emotions [53, 56], mental health outcomes [58, 59], coping strategies [54] as well as preventive and protective behaviors against COVID-19 [50, 55, 60], but negatively associated with happiness [57], psychological well-being [54], as well as self-efficacy and general mental health [20].

Interestingly, based on an earlier scale aimed at assessing SARS-related risk perception [47], Yıldırım and Güler [20] developed the CPRS, a measure of perceived risk related to the COVID-19. The CPRS is a multidimensional questionnaire that addresses both the cognitive and emotional aspects of perceived risk. The former is related to the likelihood of contracting COVID-19 and its severity, whereas the latter refers to worry that an individual experience about contracting COVID-19 (herself/himself or family members) as well as its spreading. Other than the original study by Yıldırım and Güler [20], to date, there is no studies explicitly examining the psychometric properties of CPRS in different cultures. However, studies using the CPRS showed the usefulness of the scale in predicting well-being and mental health outcomes in different contexts such as positivity, death distress and happiness in general population [57] and stress, anxiety, depression, and fear [59], subjective well-being, and parental coronavirus anxiety [58] in healthcare professionals during COVID-19 pandemic. Overall, the scale showed good psychometric properties in the original study by Yıldırım and Güler [20], and the authors concluded that both the overall score or subscale scores of the CPRS can be used as a measure of perceived risk related to COVID-19 in both research and practice settings.

Given our interest in the perceived risk of being infected at work, in this research we adapted the content of the scale items to the work context (CPRS at work, CPRS-W; see the following Measure section). More specifically, the cognitive dimension of the CPRS-W reflected the perceived likelihood of contracting COVID-19 at work, as well as its severity. Similarly, the emotional dimension subsumed the worry of a worker that she/he or a member of her/his team will contract COVID-19 at work, as well as its spreading in the work context. Accordingly, the first aim of this study was to assess the psychometric properties of the CPRS-W. More specifically, we expected a two-factor structure as originally presented, as well as adequate results in terms of construct validity (i.e., convergent and discriminant validity) and reliability [61].

### Job demands and resources related to safety at work

Building on the JD-R [21–23] and its theoretical adaptation to workplace safety [17, 24, 25], we propose in this study that the perceived risk of being infected at work (i.e., a job demand) may have negative consequences on employees’ health and well-being. Similarly, we also propose that characteristics of the job (both physical and psychosocial) that help workers to reduce or manage the risk of being infected at work can be conceived as job resources, which may be negatively associated with negative outcomes as well as affect the association between perceived risk and these outcomes. Accordingly, the second aim of this study was to investigate the association between the perceived risk of being infected at work and emotional exhaustion. We also examined the association between job resources related to safety at work and emotional exhaustion, as well as the moderating role of these job resources in the relationship between perceived risk and emotional exhaustion.

In line with the JD-R model, job characteristics can be classified either as job demands or job resources. Job demands refer to those aspects of a job (physical, psychological, social, or organizational) that require sustained physical and/or psychological (cognitive and emotional) effort from the worker and hence are associated with certain physiological and/or psychological costs. Job resources are those aspects of a job (physical, psychological, social, or organizational) that are functional in achieving work goals, reduce job demands and the associated costs (physiological and psychological), or promote personal growth, learning, and development [21, 62]. Job demands and job resources respectively trigger two different processes: a health impairment process and a motivational process. In the health impairment process, poorly designed jobs or chronic job demands require efforts and drain employees’ mental and physical resources, thus leading, over time, to exhaustion and health complaints [23, 63]. According to the motivational process of the JD-R, job resources may play a motivational role (either intrinsic or extrinsic), thus leading to high work engagement and better job performance [23, 64]. Furthermore, a lack of job resources precludes that job demands are met and that work goals are reached, which may result in job burnout [65–67]. In addition, in line with the buffer hypothesis of the JD-R, job resources may buffer the association between job demands and negative outcomes including job burnout [21, 68–70]. More specifically, by definition, job resources may reduce job demands and the psychological/physiological costs associated with them, so that the relationship between job demands and burnout will be particularly strong when job resources are low [68].

The JD-R is a flexible model that has been applied to several work-related areas, including work-home interference [71], career development [72], work ability [73], workplace bullying [74], and also safety at work [17, 24, 25]. In their seminal work about the JD-R and workplace safety, Nahrgang et al. [25] proposed that working conditions such as risks and hazards or task complexity may be conceptualized as job demands, whereas working conditions such as knowledge of safety (e.g., policies and procedures) or social support may be categorized as job resources. Furthermore, the authors also proposed that job demands related to safety deplete employees’ psychological as well as physical resources and should result in job burnout, whereas safety-related job resources may be motivating (intrinsically or extrinsically), thus being positively associated with engagement (e.g., engagement in safety activities, compliance) but negatively associated with job burnout. Finally, job burnout should be positively related to safety outcomes (e.g., accidents and injuries, unsafe behavior), whereas engagement should be negatively related to these outcomes.

In line with transactional models of stress [75, 76], which have also been applied to the JD-R [18], in this study we focused on perceived risk of being infected at work as a job demand, given the central role of individual appraisals in initiating the stress process [75]. According to the health impairment process of the JD-R, the perceived risk of being infected at work requires efforts (e.g., to handle risks) and drains employees’ mental and physical resources (e.g., time and energies), thus leading, over time, to emotional exhaustion. Previous research has considered perceived risk as a job demand in the framework of the JD-R [41, 77, 78]. Also, empirical studies, which mainly focused on conventional risks, supported the idea of an association between perceived risk (as a job demand) and negative outcomes for the individual, such as job burnout and low job satisfaction [25, 40, 41]. Overall, based on the aforementioned arguments and the results of previous research, we hypothesized that the perceived risk of being infected at work is positively associated with emotional exhaustion (hypothesis 1).

Hypothesis 1 (H1): The perceived risk of being infected at work is positively associated with emotional exhaustion.

According to the JD-R, job resources may be negatively related to job burnout [27, 66]. Moreover, in line with the buffer hypothesis of the JD-R, job resources may offset the negative impact of job demands on burnout [68]. Considering the complexity and the distinctiveness of each occupational setting [68] and given that each organization can implement different strategies to mitigate COVID-19 risk [16], in this study we focused on several job resources. These were safety systems, communication, decision-making, situational awareness, fatigue management, and participation in decision-making.

In our research, safety systems refer to the perceived quality and effectiveness of policies, procedures, or interventions implemented by an organization to improve safety outcomes with respect to the COVID-19 risk [79, 80]. For the definition of communication, decision-making, situational awareness, and fatigue management we build on the theoretical framework about non-technical skills (NTS) developed by Mariani et al. [81]. In the original model, NTS referred to workers’ cognitive, social, and personal abilities, complementary to technical skills, that may contribute to safe and efficient performance [81]. However, in this study the focus was on job resources (rather than individual skills) related to COVID-19 risk. Accordingly, the definition and the operationalization of the above-mentioned skills were adapted to reflect the ability of the whole organization as well as its members (e.g., employees and managers) to adopt and support specific behaviors concerning the risk of COVID-19 infection. More specifically, communication is a central element for teamwork and workplace safety that refers to the exchange (among colleagues and supervisors) of information, feedback or possible reactions concerning COVID-19 risk. Decision-making is the process of reaching a judgement or selecting an option, whereas situational awareness consists of constant monitoring in the workplace, observing what is happening and detecting possible relevant changes in the environment with respect to COVID-19 risk. Fatigue management refers to the identification of antecedents and consequences of fatigue (mental and physical) related to protective behaviors at work as well as the implementation of coping strategies. Finally, participation in decision making can be defined as the extent to which an organization and its managers encourage employee contribution to organizational decisions [82].

Overall, from a theoretical standpoint, we believe that all the safety-related job characteristics mentioned above can be conceived as job resources, given that they may play a motivational role (e.g., by being functional in achieving work goals, in terms of both productivity and safety outcomes). These job aspects may also be useful to reduce the perceived risk of being infected at work and the associated psychological/physiological costs (e.g., by helping workers to cope effectively with the perceived risk of contracting COVID-19) [17, 24, 25, 80].

Past research on the JD-R applied to workplace safety provided some support for the association between job resources and negative outcomes, such as emotional exhaustion and low job satisfaction, as well as the moderating role of job resources in the relationship between perceived risks and the above-mentioned outcomes [25, 40, 41]. For example, the meta-analysis by Nahrgang et al. [25] showed that job resources related to safety at work (e.g., knowledge, social support, and safety climate) were negatively associated with job burnout. Furthermore, Nielsen et al. [41] found that psychological safety climate (considered as a job resource) moderated the relationship between risk perception (conceived as a job demand) and job satisfaction in a sample of Norwegian offshore workers. Similarly, in addition to the JD-R, other theoretical models proposed that job resources attenuate the association between job demands and negative outcomes. For example, according to the Job Demand–Control–Support model [83, 84], job control and social support buffer the negative effects of job demands on employees’ well-being [85]. When applied to safety at work, job demands and control may reflect for example safety-related situational constraints and control over safety practices and procedures, respectively, whereas social support may include both perceived support at individual-level (e.g., support provided by one’s co-workers or supervisor) as well as safety climate at the group-level [86, 87]. Although empirical findings are still limited, a study among blue-collar workers by Snyder et al. [86] showed that safety control buffered the negative effects of situational constraints, in terms of workplace injuries. Moreover, in a diary study among school teachers, Garrick et al. [88] found that psychosocial safety climate moderated the relationships between daily job demands and fatigue. Hence, based on these arguments and given the assumptions of the JD-R, we hypothesized that job resources, in terms of safety systems, communication, decision-making, situational awareness, fatigue management, and participation in decision-making, are negatively associated with emotional exhaustion (hypothesis 2).

Hypothesis 2 (H2): Job resources are negatively associated with emotional exhaustion.

Furthermore, we also hypothesized that the above-mentioned job resources moderate the association between perceived risk of being infected at work and emotional exhaustion, with this association being stronger for workers with lower resources (hypothesis 3).

Hypothesis 3 (H3): Job resources moderate the association between the perceived risk of being infected at work and emotional exhaustion.

**Fig 1:**
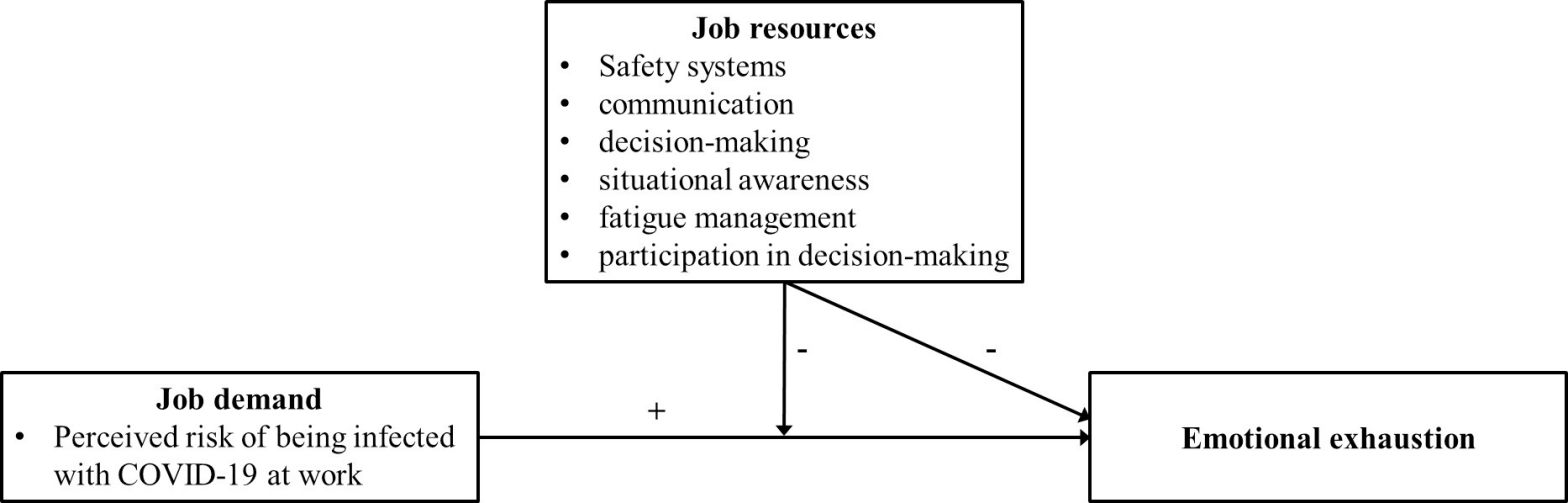
An application of the job demands-resources model to workplace safety during the COVID-19 outbreak.

The conceptual model is depicted in Fig 1.

Finally, it should be noted that demographic characteristics such as gender, age, and education may be associated with job burnout and emotional exhaustion (i.e., the dependent variable in this study) [89–95]. With respect to gender, previous research indicated that women are more likely to report burnout than men, especially with respect to emotional exhaustion [89, 90]. This may reflect differences in gender roles, with women being particularly at risk of work-family conflict–to which emotional exhaustion is strongly and reciprocally associated–due to the combined presence of demands from work and home [90]. Previous studies also showed an association between age and job burnout, although results have not been conclusive. For example, burnout and its components take time to develop, and it is possible that a positive association between age and burnout exists, with burnout reflecting the cumulated effect of prolonged or chronic stress at the end of one’s career [91]. Similarly, burnout and its component may be negatively associated with age, since employees develop coping skills with age or experience, or alternatively because employees with high levels of burnout would leave their work at a younger age [92]. Notably, the association between age and burnout could be different across gender [91, 93]. Finally, past research has suggested a negative association between education and job burnout, with lower education level being associated with higher burnout scores [94, 95]. It is possible that employees with higher education may have more opportunities to find a job characterized by higher levels of job resources (e.g., skill variety), which may result in increased motivation and reduced burnout [95]. Given the possible association between gender, age, and education on the one hand, and emotional exhaustion on the other, the hypotheses were tested both controlling for and not controlling for these demographic characteristics.

Overall, we believe that this study may contribute to fill a gap in the literature for at least three reasons. First, although several adaptations of the JD-R model to the field of workplace safety currently exist in the literature [17, 24, 25], our study was the first attempt to contextualize the JD-R to psychological safety at work during the current outbreak of COVID-19. Second, albeit the risk of infection with COVID-19 is a widespread problem, to the best of our knowledge no previous empirical research has investigated the association between the perceived risk of being infected with COVID-19 at work and employees’ health and well-being in the general working population (i.e., outside the healthcare context). Finally, as far as we know, a standardized questionnaire that assesses the perceived risk of being infected at work is currently lacking. All in all, although further research is needed to replicate and extend this study (e.g., by investigating the motivational process of the JD-R), our work may provide scholars and practitioners in the field of work and organizational psychology with useful insights and tools to understand the complex association between the perceived risk of infection (i.e., a job demand), protective factors (e.g., job resources), and psycho-physical health.

## Materials and methods

### Procedure and participants

The present study was conducted on a sample of workers from different organizations in Italy. Participants had to be employed at the time of study and were recruited through snowball sampling. Briefly, the initial participants were contacted by trained research assistants and were invited to complete an online questionnaire about their work experience. Then, participants were asked to provide contact information for other individuals potentially interested to take part in the research (e.g., colleagues), who were in turn approached by the research assistants, and so on. All the participants were informed about the general purpose of the study, that participation was confidential and voluntary, and that they could withdraw from the research at any time. Upon acceptance, they were given a link that included the informed consent form and the self-report measures. All participants had to give written informed consent before filling out the questionnaire. The research project has been approved by the Ethics Committee for the Psychological Research of the University of Padua, Italy. Data collection occurred over a period of approximately twenty days, between the end of October 2020 and the first half of November 2020. Overall, three hundred and sixty-one valid questionnaires were completed. Three participants had extensive missing data (i.e., more than 50% of missing values) and were excluded from subsequent analyses. Missing values were then estimated using the Expectation–Maximization algorithm [96, 97], and 199 missing values (1.3%) were imputed. Accordingly, the final sample comprised 358 participants. The sample consisted of 181 women (50.6%) and 177 men (49.4%) with a mean age of 36.3 years (*SD* = 12.2). Concerning education, 209 workers (58.4%) held a secondary degree, and 149 workers (41.6%) had a university degree. Regarding the type of contract, 265 workers (74%) had a permanent contract, whereas 91 (25.4%) had a temporary contract (2 missing values, 0.6%). Finally, with respect to work experience, 49.2% had been with their current company for less than 5 years and 29.1% for more than 10 years (9 missing values, 2.5%).

### Measures

The following self-report measures were administered:

The perceived risk of being infected at work was measured using an adaptation of the CPRS [20] to the work context, that is, the CPRS-W. As the original form of CPRS, the CPRS-W includes a cognitive dimension (e.g., “What is the likelihood that you would acquire the COVID-19 in your work organization?”) and an emotional dimension [e.g., “How worried are you about a member of your work team (e.g., colleagues, supervisor) contracting the COVID-19 in your work organization?”] of personal risk. The five-point response scale ranged from 1 (negligible) to 5 (very large), with higher scores indicating greater risk associated with COVID-19 in work context. In line with the authors’ suggestions [20], an overall score of perceived risk was computed.

Emotional exhaustion was assessed using the scale taken from the Italian adaptation of the Maslach Burnout Inventory—General Survey (MBI-GS) [98]. The scale was composed of nine items (e.g., “I feel emotionally drained from my work”) aimed at detecting the depletion or draining of mental resources [99]. In the present study, the six-point response scale ranged from 1 (never) to 6 (always), with higher scores referring to a high level of emotional exhaustion in work.

With respect to job resources, safety systems were measured by three items taken by the questionnaire Safety at Work (SAPH@W) [100] (e.g., “To what extent were the safety measures adopted by your Organization adequate to deal with the risk of COVID-19 contagion?”). The ten-point response scale ranged from 1 (not adequate at all) to 10 (totally adequate). Communication, decision-making, situational awareness, and fatigue management (four items each) were measured by using the respective scales taken by the SAPH@W [100]. The scales reflect the ability of the work Organization and its members (e.g., colleagues and supervisors) to adopt specific behaviors concerning the risk related to COVID-19 infection. Sample items are “Communicate effectively with superiors about the COVID-19 risk” for communication, “Make decisions quickly” (concerning the risk related to COVID-19 infection) for decision-making, “Identify the specific risks of contagion for your job” for situational awareness, and “Implement strategies to cope with physical fatigue" (due to specific behaviors such using protective devices or keeping social distancing) for fatigue management. The five-point response scale ranged from 1 (not at all) to 5 (completely). Finally, participation in decision-making was assessed by using a scale taken from the Q_u_-Bo test [101], an instrument standardized for the Italian context. The scale was composed of three items (e.g., “In my work team workers are involved in the definition of objectives and decision-making processes”), and the six-point response scale ranged from 1 (strongly disagree) to 6 (strongly agree). These scales are intended to measure conceptually distinct, although possibly related, positive aspects of the job, that is, job resources. For all these scales, higher scores referred to a high level of job resources.

### Data analysis

Prior to estimating the regression models aimed at hypothesis testing, several confirmatory factor analyses (CFA) were carried out to evaluate the psychometric properties of the self-report questionnaires that were specifically adapted or developed to measure aspects related to the risk of COVID-19 infection. These were the CPRS-W and the job resources scales. In order to evaluate model-fit for CFAs, the Satorra-Bentler scaled test statistic (χ^2^) was used [102]. Since the χ^2^ is affected by sample size, additional fit indices were considered: the root mean square error of approximation (RMSEA), the comparative fit index (CFI), and the standardized root mean square residual (SRMR). A model shows a good fit to data if the χ^2^ is non-significant. Additionally, values close to or smaller than 0.08 for RMSEA and SRMR, as well as values close to or greater than 0.90 for CFI, indicate an acceptable fit [61]. Construct validity (i.e., convergent and discriminant validity) and reliability were assessed using the average variance extracted (AVE) [103] and coefficient ω [104], respectively. AVE can be used to assess both convergent and discriminant validity. An AVE greater than .50 suggests adequate convergent validity. Furthermore, discriminant validity is established if the AVE of any two constructs is greater than their squared correlation (i.e., shared variance) [103]. For ω, values greater than .70 suggest satisfactory reliability [105].

Then, the hypothesized relationships were tested using moderated multiple regression analyses following the procedure outlined by Aiken and West [106]. Overall, six different models were estimated. In model 1 (M1), emotional exhaustion was the dependent variable, whereas the perceived risk of being infected at work and safety systems were the independent and the moderating variables, respectively. The other models were similar, except that communication (model 2, M2), decision-making (model 3, M3), situational awareness (model 4, M4), fatigue management (model 5, M5), and participation in decision-making (model 6, M6) were the moderating variables, respectively. In all the models tested, the scores of both the independent and the moderating variables were centered, and the cross-product of centered variables was entered in each model. If a significant interaction was found, the models were estimated both including and omitting the respective interaction term, to assess the additional variance explained by each of them. Then a simple slope analysis was conducted to determine whether perceived risk was associated with emotional exhaustion at high (+1*SD*) and low (−1*SD*) levels of the respective job resources. Finally, to interpret the nature of the moderating effects, significant interactions were presented graphically, following the procedure outlined by Aiken and West [106].

Because previous studies have suggested that demographic characteristics such as gender, age, and education may be associated with job burnout and emotional exhaustion [89–95] as well as perceived risk of being infected [50, 55, 60], all the regression models were estimated both controlling for and not controlling for these demographic characteristics. Given that the results were very similar, only the more parsimonious models (i.e., without controlling for the effect of gender, age, and education) were presented [107]. The models including the control variables are available in a (S1 Table). Statistical analyses were carried out using the software R version 3.6.2 [108], and, more specifically, CFAs were carried out using the lavaan package version 0.6–5 [109] for R software. Finally, AVE and coefficient ω were computed using the semTools package version 0.5–4 [110] for R software.

## Results

### Confirmatory factor analysis

First, a CFA was carried out to investigate the psychometric properties of the CPRS-W. In this model, the cognitive and the emotional dimensions of the perceived risk of being infected at work were measured by four items each. The fit indices showed a less than adequate fit to data: χ^2^(19) = 99.19, *p* < .001; RMSEA = .109, CFI = .941, SRMR = .065. Although the use of RMSEA to assess model fit in models with small degrees of freedom could be problematic (unless the sample size is very large) [111], we examined the modification indices to gain a better understanding of the potential sources of misfit. A closer inspection revealed that an error covariance (between item 7 and item 8, which shared a similar wording in our revised form of the CPRS-W) should be freely estimated. A new CFA was performed, and fit indices showed an acceptable fit to data: χ^2^(18) = 60.69, *p* < .001; RMSEA = .081, CFI = .969, SRMR = .052. AVE was .56 and .76 for the cognitive and emotional dimension, and coefficient ω was .81 and .90, respectively. The correlation between the two latent dimensions was .71, and AVE for each subscale was higher than their squared correlation. Furthermore, to support the use of an overall perceived risk score, a second-order CFA model was estimated, in which a second order-factor (i.e., the perceived risk of being infected at work) accounted for the correlations among the first-order factors (i.e., the cognitive and emotional dimensions of perceived risk). In this model, both the cognitive (β = .89, *p* < .001) and the emotional (β = .80, *p* < .001) dimensions of perceived risk loaded strongly onto the second-order factor (i.e., the perceived risk of being infected at work), which accounted for 80% and 64% of the variance in the first-order factors, respectively. Overall, the CPRS-W showed adequate psychometric properties, in terms of factor structure, construct validity (i.e., convergent and discriminant validity) and reliability. Finally, the results of the second-order CFA model supported the use of an overall perceived risk score.

Then, the psychometric properties of the scales aimed at determining job resources related to the risk of COVID-19 infection were investigated. In a six-factors model, the four dimensions of communication, decision-making, situational awareness, and fatigue management were measured with four items each, whereas both safety systems and participation in decision-making were measured with three items. The fit indices showed an adequate fit to data: χ^2^(194) = 549.65, *p* < .001; RMSEA = .072, CFI = .942, SRMR = .047. However, a closer inspection of the modification indices revealed that an error covariance between two items of fatigue should be freely estimated. These items shared a similar wording, given that they both refer to strategies implemented by the work organization to cope with employees’ fatigue (mental *vs* physical, respectively). A revised six-factor model was then estimated, and fit indices showed an acceptable fit to data: χ^2^(193) = 409.67, *p* < .001; RMSEA = .056, CFI = .965, SRMR = .047. The AVE ranged from .64 (safety systems) to .81 (fatigue management), whereas coefficient ω ranged from .84 (safety systems) to .92 (decision-making and fatigue management). The correlation between the latent factors ranged from .39 (between safety systems and participation in decision-making) to .79 (between decision-making and situational awareness), and AVE for each pair of latent factors was greater than their squared correlation. Finally, we also estimated a model in which all the items concerning job resources loaded on a single factor, and the fit indices showed a poor fit to data: χ^2^(208) = 1816.69, *p* < .001; RMSEA = .147, CFI = .738, SRMR = .092. Moreover, the proposed six-factor model had a better fit to data than the single-factor model, Δχ^2^(15) = 921.6, *p* < .001. Overall, the job resources scales showed good psychometric properties in terms of factor structure, construct validity (i.e., convergent and discriminant validity), and reliability.

As a final step, two additional models were estimated: a model in which all the items of the scales administered in the study loaded on their respective factor (i.e., a nine-factor model), and a model in which all the scale items loaded on a single factor (i.e., a single-factor model). For the nine-factor model, the fit indices showed an acceptable fit to data: χ^2^(664) = 1254.98, *p* < .001; RMSEA = .050, CFI = .940, SRMR = .051. On the contrary, the fit indices showed a poor fit to data for the single-factor model: χ^2^(700) = 5119.97, *p* < .001; RMSEA = .133, CFI = .549, SRMR = .154. Finally, the nine-factor model had a better fit to data than the single-factor model, Δχ^2^(36) = 2533.10, *p* < .001, thus further supporting the idea that the scales adopted in this study measured distinct constructs.

### Hypothesis testing

Descriptive statistics, correlations between study variables, as well as Cronbach’s alphas, are reported in Table 1. All variables had univariate skewness and kurtosis that fell within the acceptable range of ±2.0 and ±7.0, respectively [112]. Interestingly, there was a positive, medium-sized correlation between emotional exhaustion and the perceived risk of being infected at work (*r*_356_ = .35, *p* < .001). Furthermore, there were negative, small- to medium-sized correlations [113] between emotional exhaustion on the one hand, and safety systems (*r*_356_ = -.30, *p* < .001), communication (*r*_356_ = -.27, *p* < .001), decision-making (*r*_356_ = -.34, *p* < .001), situational awareness (*r*_356_ = -.25, *p* < .001), fatigue management (*r*_356_ = -.25, *p* < .001), and participation in decision-making (*r*_356_ = -.34, *p* < .001), on the other.

**Table 1:** Means, standard deviations, cronbach’s alphas, and correlations between study variables (*N* = 358).

Variable	*M*	*SD*	1	2	3	4	5	6	7	8
1. Perceived risk	2.45	0.84	(.90)							
2. Emotional exhaustion	2.61	1.14	.35***	(.92)						
3. Safety systems	7.11	2.02	-.36***	-.30***	(.84)					
4. Communication	3.58	0.97	-.20***	-.27***	.52***	(.90)				
5. Decision-making	3.27	1.00	-.22***	-.34***	.62***	.60***	(.92)			
6. Situational awareness	3.21	1.00	-.28***	-.25***	.62***	.58***	.72***	(.91)		
7. Fatigue management	2.56	1.08	-.13*	-.25***	.46***	.52***	.64***	.65***	(.95)	
8. Participation	3.41	1.38	-.10	-.34***	.38***	.48***	.54***	.45***	.50***	(.84)

*Note*: Internal consistency of the scales (Cronbach’s alpha) is displayed in the diagonal of the correlation matrix within parentheses.

* *p* < .05.

*** *p* < .001.

Results of the moderated regression analyses for safety systems (M1), communication (M2), and decision-making (M3) are presented in Table 2, whereas the moderated regression analyses for situational awareness (M4), fatigue management (M5), and participation in decision-making (M6) are displayed in Table 3. In all these models, after controlling for the effect of each specific job resource and the respective interaction term, perceived risk was positively associated with emotional exhaustion. Similarly, in all the models job resources were negatively associated with emotional exhaustion, after controlling for the effect of perceived risk and the interaction term. Hence, H1 and H2 were supported.

**Table 2:** Results from moderated multiple regression analyses: Model 1, Model 2, and Model 3 (*N* = 358).

	Model 1		Model 2		Model 3	
Dependent variable: emotional exhaustion	*B*	*SE*	*B*	*SE*	*B*	*SE*
Perceived risk	0.36***	0.07	0.42***	0.07	0.40***	0.07
Safety systems	-0.11***	0.03				
Communication			-0.25***	0.06		
Decision-making					-0.30***	0.06
Perceived risk x safety systems	-0.08*	0.03				
Perceived risk x communication			-0.17**	0.07		
Perceived risk x decision-making					-0.14*	0.06
Total *R*^2^	.17***		.18***		.20***	
Change in *R*^2^	.01*		.02**		.01*	
Simple slope low (-1*SD*)	0.52***	0.10	0.59***	0.10	0.54***	0.10
Simple slope high (+1*SD*)	0.19	0.10	0.25**	0.09	0.26**	0.09

*Note*. The moderating variable was safety systems in Model 1, communication in Model 2, and decision-making in Model 3. *B* = unstandardized regression coefficient; *R*^2^ = squared multiple correlation; *SD* = standard deviation; *SE* = standard error.

* *p* < .05.

** *p* < .01.

*** *p* < .001.

**Table 3:** Results from moderated multiple regression analyses: Model 4, Model 5, and Model 6 (*N* = 358).

	Model 4		Model 5		Model 6	
Dependent variable: emotional exhaustion	*B*	*SE*	*B*	*SE*	*B*	*SE*
Perceived risk	0.42***	0.07	0.42***	0.07	0.43***	0.06
Situational awareness	-0.18**	0.06				
Fatigue management			-0.22***	0.05		
Participation					-0.25***	0.04
Perceived risk x situational awareness	-0.10	0.06				
Perceived risk x fatigue management			0.05	0.06		
Perceived risk x participation					-0.09*	0.04
Total *R*^2^	.15***		.16***		.22***	
Change in *R*^2^	-		-		.01*	
Simple slope low (-1*SD*)	-	-	-	-	0.55***	0.09
Simple slope high (+1*SD*)	-	-	-	-	0.31***	0.09

*Note*. The moderating variable was situational awareness in Model 4, fatigue management in Model 5, and participation in Model 6. *B* = unstandardized regression coefficient; Participation = participation in decision-making; *R*^2^ = squared multiple correlation; *SD* = standard deviation; *SE* = standard error.

* *p* < .05.

** *p* < .01.

*** *p* < .001.

With respect to H3, as shown in Table 2, the interaction between perceived risk and safety systems was significant in M1, and accounted for an additional 1.3% of the variance in emotional exhaustion, *F*_change_(1, 354) = 5.51, *p* = .02. An analogous pattern of results was found for both communication and decision-making. In M2, the interaction between perceived risk and communication was significant and accounted for an additional 1.6% of the variance in emotional exhaustion, *F*_change_(1, 354) = 6.85, *p* < .01. Similarly, the interaction between perceived risk and decision-making was significant in M3, and accounted for an additional 1.1% of the variance in emotional exhaustion, *F*_change_(1, 354) = 4.86, *p* = .03.

Furthermore, as shown in Table 3, the interaction between perceived risk and participation in decision-making was significant in M6, and accounted for an additional 0.9% of the variance in emotional exhaustion, *F*_change_(1, 354) = 4.23, *p* = .04. Conversely, the interaction between perceived risk and situational awareness (M4), as well as the interaction between perceived risk and fatigue management (M5), were not significant.

**Fig 2:**
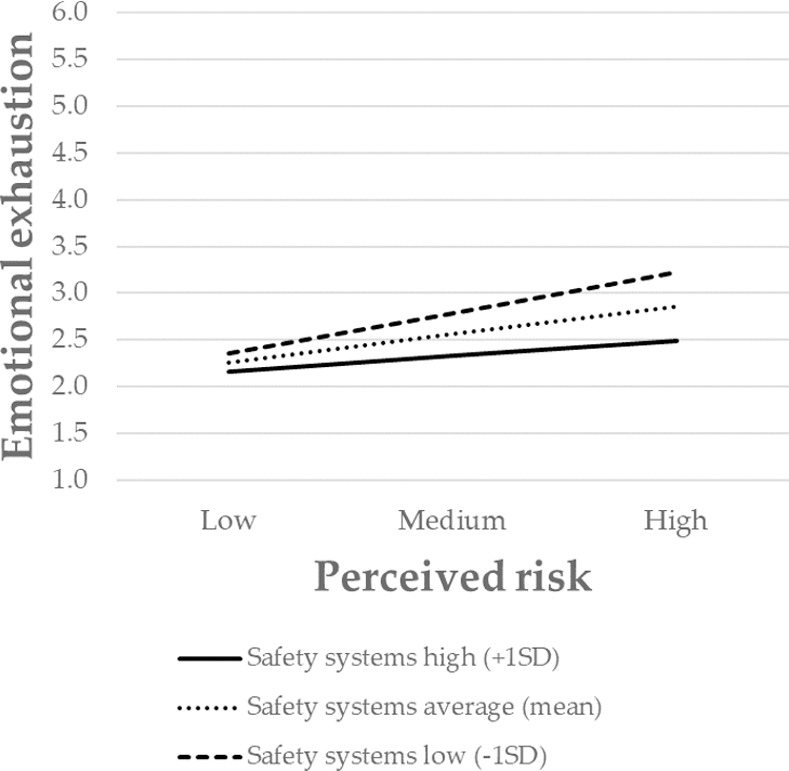
The moderating role of safety systems in the relationship between the perceived risk of being infected at work and emotional exhaustion. Perceived risk = perceived risk of being infected at work. SD = standard deviation.

**Fig 3:**
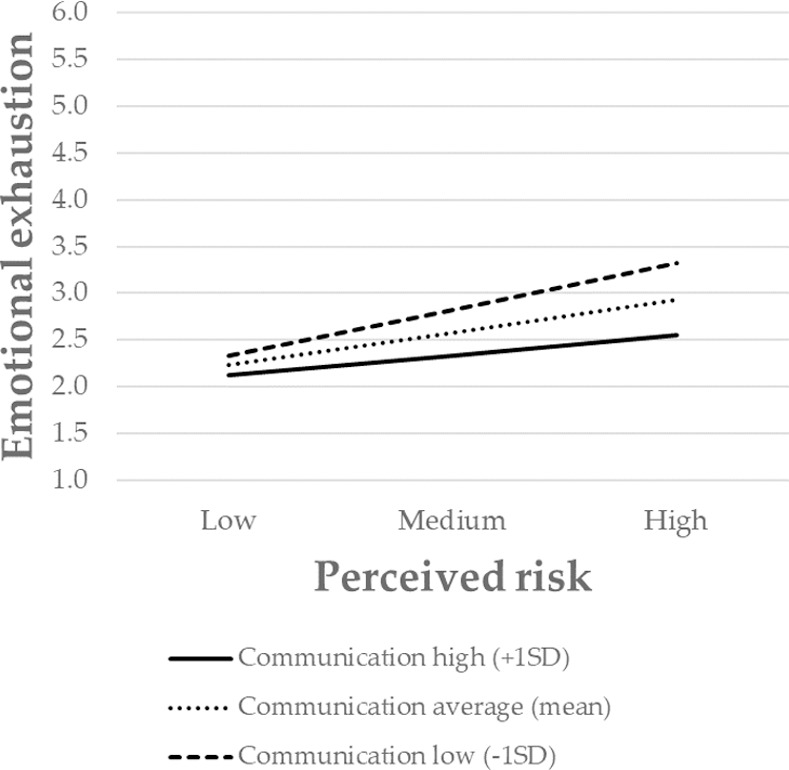
The moderating role of communication in the relationship between the perceived risk of being infected at work and emotional exhaustion. Perceived risk = perceived risk of being infected at work. SD = standard deviation.

**Fig 4:**
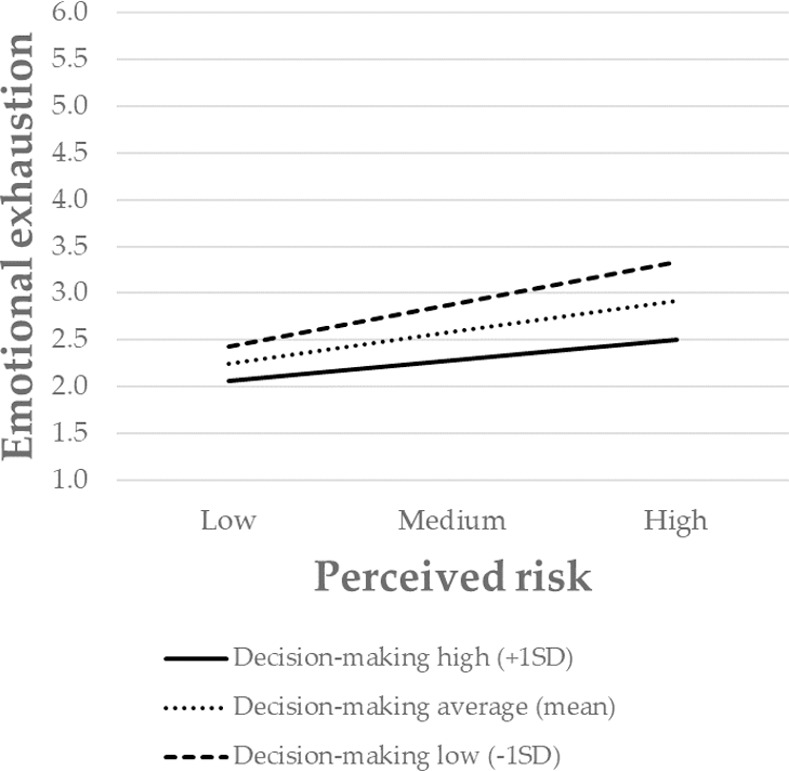
The moderating role of decision-making in the relationship between the perceived risk of being infected at work and emotional exhaustion. Perceived risk = perceived risk of being infected at work. SD = standard deviation.

**Fig 5:**
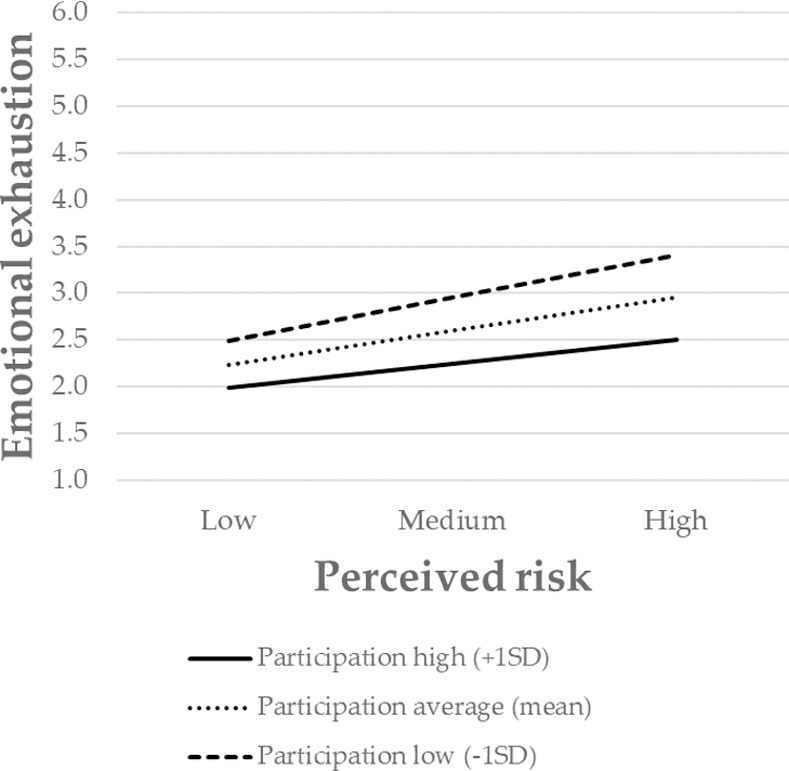
The moderating role of participation in decision-making in the relationship between the perceived risk of being infected at work and emotional exhaustion. Perceived risk = perceived risk of being infected at work. Participation = participation in decision making. SD = standard deviation.

Finally, to further interpret the nature of the moderating effect, the significant interactions were presented graphically. The association between perceived risk and emotional exhaustion was stronger for individuals with low levels of safety systems, and weaker for those with high levels of safety systems (Fig 2). The same pattern also occurred for communication (Fig 3), decision-making (Fig 4), and participation in decision-making (Fig 5). Overall, H3 was partially supported.

## Discussion

The ongoing outbreak of COVID-19 is affecting many areas of everyday life, including family, education, and work [10–12], with major health (physical and mental), social, and economic consequences [6–9]. Regarding the work context, both physical and psychological safety play a central role for workers and organizations during this pandemic and should be considered jointly [17]. Hence, in a perspective of prevention and occupational health promotion, it is important to consider those aspects of the job and the work environment that may affect both physical and psychological outcomes related to COVID-19 risk. These refer, for example, to the physical consequences of COVID-19 infection as well as work-related stress/job burnout due to the risk of being infected at work, respectively.

In this study we specifically focused on the latter. Building on the JD-R [21–23] and its theoretical adaptation to workplace safety [17, 24, 25], we proposed that the perceived risk of being infected with COVID-19 at work can be conceptualized as a job demand (i.e., a risk factor for work-related stress), whereas those physical and psychosocial characteristics of the job that help workers to reduce or manage the risk of being infected at work can be conceived as job resources (i.e., protective factors) [19]. Accordingly, based on the JD-R, we hypothesized that the perceived risk of being infected at work is positively associated with emotional exhaustion. We also hypothesized that job resources related to safety at work, in terms of safety systems, communication, decision-making, situational awareness, fatigue management, and participation in decision-making, are negatively associated with emotional exhaustion. Finally, in line with the buffer hypothesis of the JD-R, we hypothesized that the aforementioned job resources attenuate the association between the perceived risk of being infected at work and emotional exhaustion [68].

The results of this study largely supported our predictions. In fact, the perceived risk of being infected at work was positively associated with emotional exhaustion, whereas job resources were negatively associated with it. Furthermore, safety systems, communication, decision-making, and participation in decision-making (but not situational awareness and fatigue management) moderated the relationship between the perceived risk of being infected at work and emotional exhaustion, with this association being stronger for workers with lower resources.

Taken together, our results are in line with the JD-R, a flexible theoretical model that can be applied to different contexts and work-related domains, including safety at work during the outbreak of COVID-19. However, as noted by Schaufeli and Taris [27], the JD-R is a general, descriptive model that specifies the relationships between classes of constructs (e.g., job demands and resources, health and motivation) without giving detailed explanations about the psychological processes involved. Therefore, in line with Bakker and Demerouti [22], we believe that the conservation of resources (COR) theory [28, 29] can provide an in-depth insight into the psychological mechanisms underlying the associations examined in this study.

According to the COR theory, individuals try to acquire, retain, and protect things that they value, that is, resources, which may include objects (e.g., house, clothes), conditions (e.g., occupational status, health), personal characteristics (e.g., occupational skills, self-esteem), or energies (e.g., time, physical/psychological energy). In this perspective, psychological stress may occur when resources are threatened with loss or actually lost, or when individuals do not gain adequate resources following relevant investment of resources. Moreover, a COR’s principle is that people have to invest resources in order to protect against loss of resource, recover from losses, and gain additional resources. This implies that individuals with greater resources are less vulnerable to resource loss, and more capable of resource gain, than those with fewer resources. Furthermore, given that the loss of resources is stressful, and because individuals have to invest resources to counterbalance further resource loss, it follows that, one initial loss have occurred, people become more and more vulnerable to ongoing loss (i.e., loss spirals).

Applied to the topic of safety at work during the outbreak of COVID-19, the COR suggests that the perceived risk of being infected at work may threaten resources that are central to individuals, such as health and work-related safety. At this stage, workers may invest additional resources at work, to protect against resource losses. For example, they may engage in coping efforts actively [114], through cognitive and behavioral strategies (e.g. monitoring the work environment to detect potential hazards, engaging in counterfactual thinking about safety-related events, adopting protecting behaviours) [25, 115–117]. This implies the investment of resources such as, for example, time, effort, and physical/psychological energies, which may result in resource depletion and, eventually, emotional exhaustion [114]. This provides theoretical support to our first hypothesis.

Conversely, job resources can replenish personal resources such as physical/psychological energies and capacities (e.g., by being functional in achieving work goals, in terms of both productivity and safety outcomes), thus preventing emotional exhaustion. Furthermore, in line with the COR, individuals with greater resources are less vulnerable to resource loss than those with fewer resources. In this perspective, workers may use resources available to them to cope effectively with the perceived risk of being infected at work, thus reducing the associated costs. This prevents further resource loss as well as negative consequences for the individual (e.g., emotional exhaustion). With respect to workplace safety during the COVID-19 outbreak, examples of resources that may be valuables for workers to achieve work goals safely and cope with the perceived risk of being infected at work are the availability of effective safety-related policies and procedures at work (i.e., safety systems), the exchange of good-quality information about COVID-19 risk across team members (i.e., communication), rapid decision-making processes that prioritize safety at work (i.e., decision-making), and the opportunity to affect the organization of one’s work (i.e., participation in decision making) [16, 41, 81, 118].

Interestingly, it should be noted that situational awareness and fatigue management did not attenuate the association between the perceived risk of being infected at work and emotional exhaustion, while being negatively associated with it. A possible explanation is that the adoption of strategies—at organizational or team level—that encourage the constant monitoring of the work environment (i.e., situational awareness) as well as an adequate management of mental and physical fatigue (i.e., fatigue management) can be useful for workers to replenish personal resources, thus preventing job burnout. For example, situational awareness and fatigue management may be valuable for employees to achieve their objectives safely (e.g., by helping them to identify possible risky situations) as well as to recover adequately from psycho-physical fatigue, respectively. However, by not being directly aimed at taking actions to manage the risk of infection at work (i.e., there is no match between job demand and job resource) [119, 120], the aforementioned resources might not be useful for workers to effectively reduce the psychological and physiological costs associated with the perceived risk. Conversely, job resources that are more specifically directed at risk management may be more valuable for workers to actively cope with the perceived risk of infection (i.e., job resource match job demands) [119], thus reducing the associated costs. This is the case, for instance, of effective and shared decision-making processes or quick and effective communications aimed at reacting readily to risky situations. Another plausible explanation is that the value of job resources depends on the work context [121]. For example, it is possible that situational awareness is especially relevant to employees with non-standardized work procedures (e.g., those who interact directly with customers), while fatigue management is particularly beneficial for workers who have to invest considerable physical and mental energies to handle the risk of contagion (e.g., healthcare employees). Clearly, although these speculations seem reasonable from a theoretical standpoint, future research should specifically address these points. Overall, although with the aforementioned exceptions, we believe that the results of this study provided theoretical support to our second and third hypothesis.

We believe that these findings provided a valuable contribution to the existing literature for several reasons. First, although researchers worldwide devoted attention to COVID-19 risk perception [20, 45–52], this study specifically focused on the perceived risk of being infected with COVID-19 at work and its negative consequences. By showing an association between perceived risk and emotional exhaustion, our research suggests that the ongoing outbreak of COVID-19 may substantially affect individuals’ well-being at work across different occupational sectors. Second, our study highlighted the importance of safety-related job resources with respect to COVID-19 risk. We believe this to be a relevant point. The risk of infection at work cannot likely be eliminated, since workplaces and the organization of work have some features that may facilitate the spread of SARS-CoV-2 [15]. Consequently, organizational- or team-level factors that can help workers to reduce or manage risks are extremely valuable. On the one hand, the adoption of different safety measures can make the work environment more stable and controllable, thus allowing workers to feel safe [41]. On the other hand, job resources may be useful for employees to achieve their objectives safely and cope effectively with the risk of infection [22]. Finally, this study contributes to the broader literature on the JD-R applied to safety at work by examining the moderating role of job resources in the relationship between perceived risk and job burnout, a pattern of associations that has been rarely examined by past research [24]. Although with some exceptions [41], previous studies and reviews [17, 25] focused on the association between perceived risks and negative/positive outcomes, such as job burnout and work engagement, but the possible moderating role of job resources was rarely taken into account.

We believe that this study outlines interesting avenues for future research. Building on the JD-R, forthcoming studies could investigate additional situational and individual factors, in terms of job and personal demands [22], that may affect the association between the perceived risk of being infected at work and emotional exhaustion. Given that the effects of job demands tend to accumulate [122], it is possible that the association between the perceived risk and emotional exhaustion is stronger when workers have to cope with several demands at a time (e.g., when individuals generally have also limited time or resources to complete their tasks or they have frequent demanding interactions with colleagues or customers). Personal demands refer to those aspects of the self that compel individuals to invest disproportionate effort in their work and/or hinder their abilities to successfully cope with their work environment [123]. Accordingly, it is possible that personal demands, such as high levels of perfectionism or need for control, may affect the way in which individuals experience or react to the perceived risk of infection at work [123, 124]. Similarly, the role of additional job and personal resources could be examined. For example, according to the triple-match principle [119], the interactive effects of job resources and job demands is particularly observed when resources, demands, and outcomes are grounded on qualitatively identical dimensions (e.g., they all pertain to emotional aspects). Accordingly, given that the emotional component is central in risk perception [125], emotional job resources (e.g., emotional support from supervisors and colleagues) [126] could be especially relevant to mitigate the effects of worry and concern about COVID-19 risk on emotional exhaustion. Similarly, with respect to personal resources, the role of emotional competences (i.e., the degree to which workers feel emotionally competent to face job demands) [127] could be investigated.

We believe that our study has relevant practical implications, in terms of primary prevention, for organizations and practitioners. First, to prevent emotional exhaustion, the perceived risk of being infected at work should be considered as an additional job demand for workers. Furthermore, previous research showed that job risks are associated with higher workload, as employees have to perform additional tasks to manage risky situations [128]. Hence, organizations should consider that workers need to invest supplementary psycho-physical energies to manage the risk of infection and provide them with adequate time and resources (e.g., equipment, adequate spaces for social distancing) to achieve their objectives safely. Second, organizations should be encouraged to optimize the balance between job demands and job resources. In this perspective, the availability of effective safety-related policies and procedures, the exchange of good-quality information about COVID-19 risk across team members, rapid decision-making processes that prioritize safety at work, and the opportunity for employees to affect the organization of one’s work may be useful for workers to reduce emotional exhaustion and cope effectively with the perceived risk of being infected at work.

Finally, this study had some limitations. First, in this study data were collected using an online questionnaire, which made it possible to access a large number of participants from different regions across Italy during the current outbreak of COVID-19. Although previous research has shown that data provided by Internet questionnaires are of at least as good quality as those provided by more traditional paper-and-pencil questionnaires [129], some drawbacks need to be acknowledged. For example, workers with higher levels of workload, and possibly with higher risk of being infected at work, may not have found the time to fill the online questionnaire. Similarly, people with less access to internet-based devices may have taken part in this research less frequently–and with greater difficulties–than others. Second, the focal constructs were determined using the same measurement method, (i.e., self-report questionnaires), and the observed relationships may be affected by common method bias [130]. Hence, future research could adopt, for example, observer-ratings of job demands and resources, at individual- or group-level (e.g., supervisors’ rating), as well as objective measures of emotional exhaustion (e.g., biomarkers of stress) [19, 131, 132]. Furthermore, the cross-sectional design of this research precluded conclusions about the direction of the observed relationships. Although our hypotheses are in line with the health impairment process of the JD-R, reversed causal and reciprocal effects are possible. For example, employees who experience emotional exhaustion may also perceive higher levels or risk (e.g., given their diminished coping resources, in terms of psycho-physical energies) and lower resources. Accordingly, future longitudinal or within-person studies could investigate these possible patterns of relationships over time. For example, in line with the JD-R model and the COR theory, future research could examine the possible reciprocal effects between emotional exhaustion and the perceived risk of infection/job resources over time (e.g., loss spirals) [114]. Moreover, a diary study could investigate the association between daily perceived risk of infection (e.g., in terms of risky situations during a workday) and daily emotional exhaustion or fatigue in the evening, as well as the moderating role of daily job resource in this relationship [133].

## Conclusions

In conclusion, this study found that the perceived risk of being infected with COVID-19 at work, conceptualized as a job demand, was positively associated with emotional exhaustion. Similarly, our study showed that job resources related to COVID-19 may reduce the psychological/physiological costs associated with the perceived risk of infection, as well as its negative outcomes. Overall, our study highlighted the importance of a good balance between job demands and job resources related to COVID-19 in preventing possible detrimental consequences for workers’ well-being, for example in terms of job burnout.

## Supporting information

S1 Table (DOCX)

S1 Dataset (TXT)

S1 File (TXT)
