# Supplementary material for: The perceived risk of being infected at work: An application of the job demands–resources model to workplace safety during the COVID-19 outbreak
Source: PLoS One. 2021 Sep 9;16(9):e0257197. doi: 10.1371/journal.pone.0257197 (PMC8428687; doi:10.1371/journal.pone.0257197)
Supplement: S1 Table — (DOCX) [file pone.0257197.s001.docx]

The models described in the manuscript were also estimated controlling for the effect gender, age, and education. Results of the moderated regression analyses for safety systems (model 1a), communication (model 2a), and decision-making (model 3a) are presented in Table 1, whereas the moderated regression analyses for situational awareness (model 4a), fatigue management (model 5a), and participation in decision-making (model 6a) are displayed in Table 2.

Table 1. Results from Moderated Multiple Regression Analyses: Model 1a, Model 2a, and Model 3a (*N* = 358).

|  | Model 1a | |  | Model 2a | |  | Model 3a | |
| --- | --- | --- | --- | --- | --- | --- | --- | --- |
| Dependent variable: emotional exhaustion | *B* | *SE* |  | *B* | *SE* |  | *B* | *SE* |
| Gender^a^ | 0.02 | 0.11 |  | 0.05 | 0.11 |  | 0.04 | 0.11 |
| Age | 0.00 | 0.00 |  | 0.00 | 0.00 |  | 0.00 | 0.00 |
| Education^b^ | 0.14 | 0.12 |  | 0.10 | 0.12 |  | 0.10 | 0.11 |
| Perceived risk | 0.36*** | 0.07 |  | 0.42*** | 0.07 |  | 0.40*** | 0.07 |
| Safety systems | -0.11*** | 0.03 |  |  |  |  |  |  |
| Communication |  |  |  | -0.25*** | 0.06 |  |  |  |
| Decision-making |  |  |  |  |  |  | -0.30*** | 0.06 |
| Perceived risk x safety systems | -0.08* | 0.03 |  |  |  |  |  |  |
| Perceived risk x communication |  |  |  | -0.17* | 0.07 |  |  |  |
| Perceived risk x decision-making |  |  |  |  |  |  | -0.14* | 0.06 |
| Total *R*^2^ | .17*** |  |  | .18*** |  |  | .20*** |  |
| Change in *R*^2^ | .01* |  |  | .01* |  |  | .01* |  |
| Simple slope low (-1*SD*) | 0.52*** | 0.10 |  | 0.58*** | 0.10 |  | 0.54*** | 0.10 |
| Simple slope high (+1*SD*) | 0.20* | 0.10 |  | 0.26** | 0.09 |  | 0.27** | 0.09 |

*Note*. The moderating variable was safety systems in Model 1a, communication in Model 2a, and decision-making in Model 3a. *B* = unstandardized regression coefficient; *R*^2^ = squared multiple correlation*; SD* = standard deviation; *SE* = standard error. * *p* < .05. ** *p* < .01. *** *p* < .001.

^a^0 = male, 1 = female.

^b^0 = secondary degree, 1 = university degree.

Table 2. Results from Moderated Multiple Regression Analyses: Model 4a, Model 5a, and Model 6a (*N* = 358).

|  | Model 4a | |  | Model 5a | |  | Model 6a | |
| --- | --- | --- | --- | --- | --- | --- | --- | --- |
| Dependent variable: emotional exhaustion | *B* | *SE* |  | *B* | *SE* |  | *B* | *SE* |
| Gender^a^ | 0.01 | 0.12 |  | 0.04 | 0.11 |  | 0.08 | 0.11 |
| Age | 0.00 | 0.00 |  | 0.00 | 0.00 |  | 0.00 | 0.00 |
| Education^b^ | 0.13 | 0.12 |  | 0.10 | 0.12 |  | 0.17 | 0.11 |
| Perceived risk | 0.42*** | 0.07 |  | 0.42*** | 0.07 |  | 0.43*** | 0.06 |
| Situational awareness | -0.18** | 0.06 |  |  |  |  |  |  |
| Fatigue management |  |  |  | -0.21*** | 0.05 |  |  |  |
| Participation |  |  |  |  |  |  | -0.26*** | 0.04 |
| Perceived risk x situational awareness | -0.10 | 0.06 |  |  |  |  |  |  |
| Perceived risk x fatigue management |  |  |  | 0.06 | 0.06 |  |  |  |
| Perceived risk x participation |  |  |  |  |  |  | -0.09* | 0.04 |
| Total *R*^2^ | .15*** |  |  | .17*** |  |  | .23*** |  |
| Change in *R*^2^ | - |  |  | - |  |  | .01* |  |
| Simple slope low (-1*SD*) | - | - |  | - | - |  | 0.55*** | 0.09 |
| Simple slope high (+1*SD*) | - | - |  | - | - |  | 0.31*** | 0.09 |

*Note*. The moderating variable was situational awareness in Model 4a, fatigue management in Model 5a, and participation in Model 6a. *B* = unstandardized regression coefficient; Participation = participation in decision-making; *R*^2^ = squared multiple correlation*; SD* = standard deviation; *SE* = standard error. * *p* < .05. ** *p* < .01. *** *p* < .001.

^a^0 = male, 1 = female.

^b^0 = secondary degree, 1 = university degree.
